# Perforated neonatal appendicitis mimicking necrotizing enterocolitis in a premature neonate: a case report and literature review

**DOI:** 10.1093/jscr/rjae471

**Published:** 2024-07-29

**Authors:** Mahmoud R Manasra, Thaer Alhroob, Mohammad Marrawani, Dalia Batanje, Yahya Imran, Moatz Harahsha, Abdul-Karim Amleh, Diaa Zugayar

**Affiliations:** Palestine Polytechnic University College of Medicine and Health Science, PO Box 198, Hebron, Palestine; Neonatal Intensive Care Unit (NICU), H-Clinic Specialty Hospital, PO Box 108, Ramallah, Palestine; Palestine Polytechnic University College of Medicine and Health Science, PO Box 198, Hebron, Palestine; Pediatric Department, Al-Makassed Islamic Charitable Society Hospital, Jerusalem 19482, Israel; An-Najah National University, PO Box 7, Shkhem, Palestine; Neonatal Intensive Care Unit (NICU), H-Clinic Specialty Hospital, PO Box 108, Ramallah, Palestine; Neonatal Intensive Care Unit (NICU), H-Clinic Specialty Hospital, PO Box 108, Ramallah, Palestine; Minimally Invasive Pediatric Surgery, Hadassah Medical Center, 91120 Jerusalem, Israel

**Keywords:** appendicitis, enterocolitis, necrotizing, neonate, preterm

## Abstract

Necrotizing enterocolitis (NEC) predominantly affects preterm infants and can mimic other conditions like acute appendicitis. Neonatal appendicitis (NA) is extremely rare, with an incidence of 0.04–0.2% and high fatality rates. Due to its rarity and resemblance to other neonatal conditions, NA diagnosis is often delayed. We report a case of a 2220-g male preterm neonate delivered at 31 + 5 weeks via urgent cesarean section due to chorioamnionitis, initially misdiagnosed with NEC but later found to have a perforated appendix. The neonate recovered well post-surgery, with the ileostomy reversed 10 weeks later. Prompt surgical intervention is crucial for NA, as it requires different management than NEC. This case underscores the importance of considering NA in preterm infants with severe abdominal symptoms and emphasizes timely surgical intervention to improve outcomes. Additionally, it supports the hypothesis that localized NEC involving the appendix may have a better prognosis than generalized NEC.

## Introduction

An inflammatory bowel condition called necrotizing enterocolitis (NEC) mainly affects preterm infants [1]. NEC's clinical manifestation can mimic several diseases with different aetiologies [1]. One of them is acute appendicitis. 141 occurrences of appendicitis in neonates were documented between 1905 and 2000 [2], making it the most prevalent surgical diagnostic for paediatric patients that necessitates hospital admission [2]. However, it is extremely uncommon in the neonatal age group. The reported incidence ranges from 0.04 to 0.2% [2–4]. Neonatal appendicitis (NA) has a significant fatality rate [3]. Appendiceal involvement is not found until after an exploratory procedure, as the diagnosis of NA is frequently overlooked before surgery. Some variables contribute to the delay in diagnosis, such as the disease's rarity and how similar it seems to other more prevalent newborn illnesses [3]. We report a case of perforated appendicitis in a preterm neonate presenting as NEC and successfully managed by surgery.

## Case report

A 2220-g male, the second part of a dichorionic-diamniotic twin as a product of *in vitro* fertilization, was born by urgent cesarean section (CS) with a gestational age of 31 + 5 weeks to a 24-year-old mother. The mother was on anticoagulant and aspirin therapy due to secondary infertility. A detailed ultrasound was done in the second trimester during the antenatal screening and showed no abnormalities. The mother had gestational diabetes mellitus, which was treated with an oral hypoglycemic agent; a urinary tract infection, which was treated with an oral antibiotic; and a premature preterm rupture of membranes (PPROM) 5 days before delivery, which was complicated by chorioamnionitis. As a result, an urgent CS was performed at our hospital.

The baby was delivered with Apgar scores of 8/10 at 1 and 5 minutes. Responding to brief positive pressure ventilation, he was admitted to the Neonatal Intensive Care Unit, where, on the first day, he was put on an O2 nasal cannula for support and started on a broad spectrum of prophylactic IV antibiotics due to the positive results of the placental swab culture for *Escherichia coli*. On the second day, after stabilization, a lumbar puncture was done, and CSF analysis showed partially treated meningitis, and antibiotics were adjusted according to culture sensitivity.

The results of the 24-hour laboratory tests showed hyperbilirubinemia, which was managed with phototherapy, and symptomatic polycythemia, for which a partial exchange transfusion was done. The baby improved clinically through the next few days of admission; he remained hemodynamically stable on room air, and tolerated oral feeding of premature milk formula or expressed breast milk with a gradual increase and weight gain until the 8th day of admission.

On the 8th day of admission, he developed severe abdominal distension, mottled skin, and a fever of 38°C. On examination, the abdomen was distended and tense on palpation, so he was put on nil per os (NPO), a nasogastric tube was placed for decompression, blood culture, arterial blood gas (ABG), CRP, CBC, stool for occult blood, and an abdominal X-ray were requested. He was started on intravenous Vancomycin, Amikacin, and Metronidazole.

**Figure 1:**
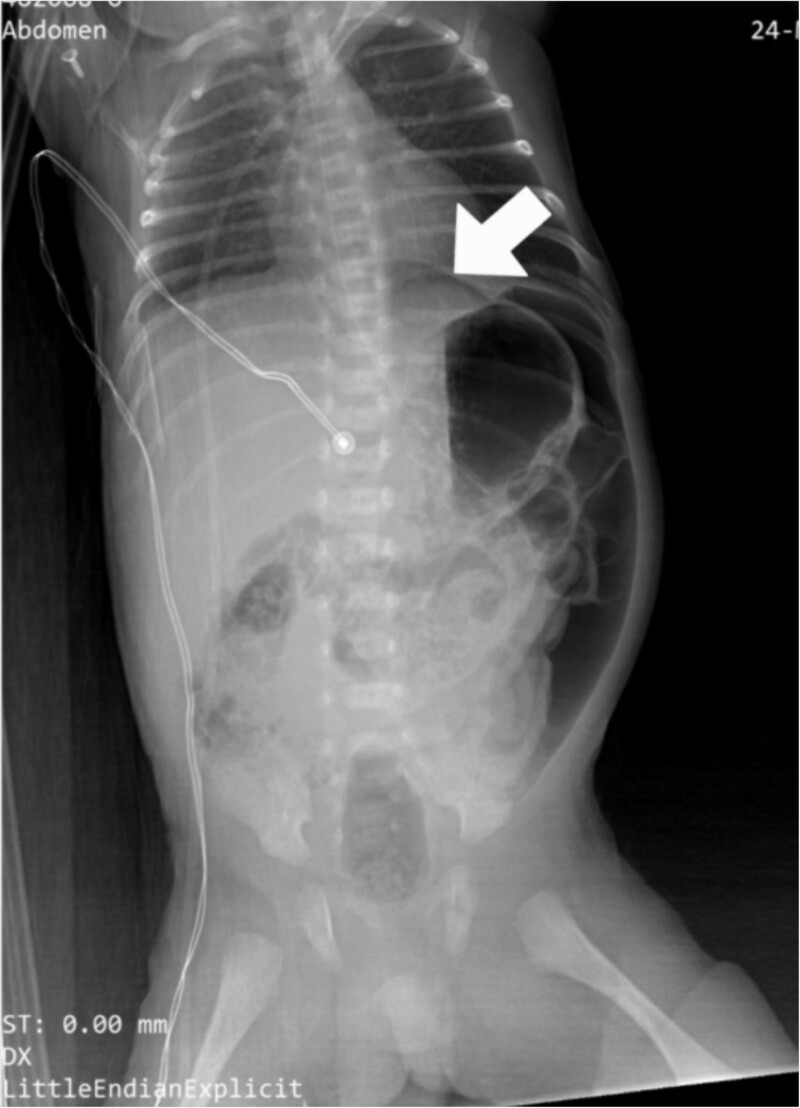
Preoperative abdominal X-ray: lateral decubitus view showing pneumoperitoneum (white arrows).

The CBC showed that the white blood cell count was 2.1 × 10^9^ cells/L, the platelet count was 276 ×10^9^ cells/L, and the CRP level was 5.2 mg/dl. There was no occult blood in the stool, the ABG showed mild metabolic acidosis with a lactate level of 3.46, and an abdominal X-ray showed pneumoperitoneum with increased abdominal distension (Fig. 1). The decision to perform an emergent exploratory transverse laparotomy was made with a presumed diagnosis of intestinal perforation as a complication of NEC.

**Figure 2:**
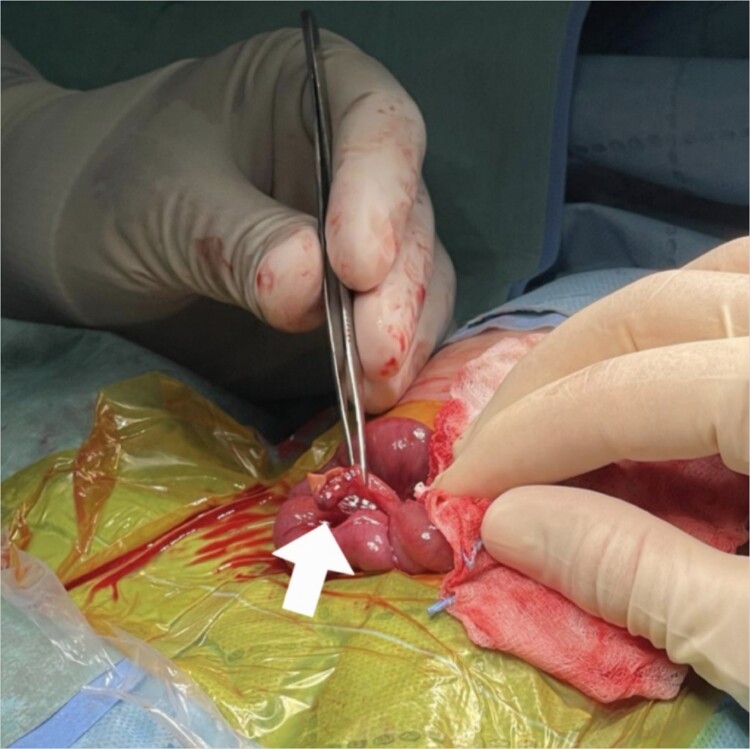
Intraoperative findings: inflamed appendix with perforation on the tip (arrow).

Intraoperative findings included viable loops of the bowel with fibrinous exudates in the right lower quadrant and an inflamed appendix with perforation on the tip (Fig. 2). Following an appendectomy, an ileostomy was created on the right lower quadrant, and an orogastric tube was inserted for gastric decompression. Pathologic analysis of the appendix revealed transmural inflammation, localized infarction, and perforation.

He was kept NPO for 7 days, then feeding was restarted gradually with good tolerability until the day of discharge. The baby increased in weight as appropriate; thus, the ileostomy was reversed 10 weeks after the surgery.

## Discussion

NA had less than 50 reported cases in the last 30 years and just more than 100 over the last century, making it a very rare condition [5]. The appendix obstruction is unlikely to occur due to the low incidence of infections predisposing to lymphadenopathy of the gut, soft diet, and recumbent position, which explains NA being uncommon [6]. It is rare to observe different localizing signs in the right upper quadrant of the abdomen, such as erythema, discomfort, and lumps [7]. Preoperative diagnosis is rarely made because of the lower incidence, non-specific clinical symptoms, and infrequent localizing signs. NA was found to be more common in boys than girls (3:1), and 25–50% of the reported cases were found in preterm neonates, with a third of cases initially diagnosed as NEC [7].

The way appendicitis appears in newborns lacks specificity and shares similarities with the presentation of NEC [8]. NEC is often misdiagnosed for neonatal perforated appendicitis (NPA) during the preoperative assessment, but diagnosis is conferred intraoperatively [9]. Abdominal distension, tenderness, feeding intolerance, and fever are the most frequent findings [2, 7]. Abdominal distension, as in our patient, was the most common reported clinical feature; according to the literature, it was reported in 89% of cases [7]. Rapid development of abdominal sepsis may be complicated by the perforation of the appendix due to a delay in diagnosis [8]. NPA is present in up to 85% of cases; pneumoperitoneum is detected in only half of them [7]. The benefit of early surgical intervention is shown by the paradoxical fact that perforation predicts much lower mortality than non-perforated patients due to timely clinical recognition [8]. An 18–28% mortality rate was reported last year’s [8].

Many pieces of literature suggest that NPA in the first weeks of life may be due to an isolated form of NEC [6]. Explaining that low immunity or sepsis (prematurity, maternal chorioamnionitis) can increase the incidence of NPA [6, 9, 10]. Perforation due to primary appendisitis cannot be histologically distinguished from isolated NEC appendicopathy [8]. In a healthy premature neonate without comorbid risk factors for appendicitis, it’s important to consider a localised form of NEC affecting the appendix as a potential differential diagnosis for NPA. We believe that our 8-day preterm neonate had an isolated form of NEC manifested as NPA, sparing small and large intestines, which were found to be healthy. In a recent study that reviewed four cases of NPA attributable to NEC, it was suggested that the prognosis for NEC specifically involving the appendix may be more favourable than that for nonspecific NEC affecting the intestines [11]. We believe that we add another case to the literature that can support this fact, as our patient's postoperative prognosis was good.
